# Distal ulna fractures in adults—subcapitular, transverse fractures did not benefit from surgical treatment

**DOI:** 10.1007/s00402-022-04336-1

**Published:** 2022-01-21

**Authors:** Maria Moloney, Simon Farnebo, Lars Adolfsson

**Affiliations:** 1 Department of Plastic Surgery, Hand Surgery and Burns, Faculty of Health Sciences, Linköping University, 58185 Linköping, Sweden; 2 Department of Orthopaedics, Linköping University, Linköping, Sweden; 3 Department of Biomedical and Clinical Sciences, Linköping University, Linköping, Sweden

**Keywords:** AO, DRUJ, Fracture, PRWE, Distal ulna, Wrist

## Abstract

**Introduction:**

Fractures of the distal ulna, excluding the styloid, are rare. The cause of injury is often a fall on an outstretched hand with an extended wrist, and in most cases there is a concomitant distal radius fracture. The aims of this retrospective study were to investigate the results of the current treatment of distal ulna fractures in adults, with or without a concomitant distal radius fracture, and if a recently presented fracture classification could predict outcome.

**Materials and methods:**

Patients, 18 years or older, treated for a fracture of the distal third of ulna in our county, were included. Fractures of the styloid tip were excluded. The radiographs of the fractures were independently classified by two specialists in radiology according to the 2018 AO/OTA classification. Follow-up was performed 5–7 years after the injury, through the questionnaire Patient-Rated Wrist Evaluation (PRWE) and new radiographs of both wrists.

**Results:**

Ninety-six patients with 97 fractures were included and filled out the PRWE. 65 patients also had new radiographs taken. 79 patients were women and the mean age at the time of injury was 63 years (SD 14.5). The most common fracture class was the extra-articular transverse fracture, 2U3A2.3 (42%). We found that 40% of the fractures had been treated by internal fixation and only 2 fractures had not healed, one conservatively treated and one operated. The median PRWE was 15 (IQR 33.5). The PRWE score was significantly worse in the operated ulna fractures (*p* = 0.01) and this was also true for extra-articular transverse fractures 2U3A2.3 (*p* = 0.001). Initial displacement was more common in operated transverse fractures, but it could not be proven that this was the reason for the inferior result.

**Conclusions:**

Distal ulna fractures almost always unite and the result is comparable to that of isolated distal radius fractures when measured by PRWE. Based on the opinions of the radiologists and how often a consensus discussion was needed for classification, we found the updated AO classification system difficult to use, if dependent only on standard radiographic views. In the present study, transverse extra-articular ulna fractures did not benefit from internal fixation regardless if associated with a distal radius fracture or isolated.

## Introduction

Falling over on an outstretched hand with an extended wrist is a common cause of injury seen in every emergency department. This trauma can cause a multitude of injuries in the wrist, most commonly fractures of the distal radius. Fractures of the distal ulna are less common, but are often associated with distal radius fractures, most often in the form of fractures of the ulnar styloid. Even rarer are fractures of the ulnar head and neck, with an incidence of 15/100.000 person-years [13]. Inappropriate treatment of distal ulna fractures may lead to non-union, bridging callus between distal radius and ulna and an altered relationship in the distal radioulnar joint (DRUJ), resulting in joint incongruence and instability. A malunited fracture may also cause ulnar-sided wrist pain and limited forearm rotation [2]. In rotation of the forearm the radiocarpal unit rotates around the fixed and stable ulna, with its large articular surface [4]. The ulnar head has approximately 270° of articular cartilage making internal fixation difficult [15]. The significance of distal ulna fractures may be underestimated since not much interest has been shown for their incidence, treatment or functional results; a review of the literature in 2008 concluded that there is little scientific support to guide the management [6].

The aims of this retrospective study were to investigate the results after distal ulnar fractures, excluding the styloid, with or without a concomitant distal radius fracture, and if a recently presented fracture classification could be used to predict outcome.

## Methods

The study protocol was reviewed and approved by the Regional Ethical Review Board (Dnr 2014/200-31).

### Patients

Data from all patients in our county treated for a fracture of the distal ulna, isolated or in combination with a fracture of the distal radius, in 2010–2014, were collected. All patients who had visited one of the three orthopaedic departments in the area received an ICD-10 diagnosis code in the digital journal system. In 2015, we searched the central database for all codes of a distal forearm fracture (S52.50, S52.51, S52.60, S52.61, S52.20, S52.21, S52.80, S52.81) during 2010–2012 and in 2019, we extended the search to include fractures sustained during 2013 and 2014. The radiographs of all patients that had received one of these codes were screened in the digital radiology system PACS/IDS7 to identify all who had suffered a fracture of the distal third of the ulna during the defined time period. Patients under the age of 18 and those having sustained a fracture of only the ulnar styloid tip (with or without concomitant radius fracture) were excluded from the study, as well as patients who were deceased or had emigrated from Sweden.

Patients with fractures sustained 2010–2012 were included in 2017, and those with fractures sustained 2013–2014 during 2019–2020. The identified individuals were approached by regular mail in which they were offered to participate in the study. Enclosed were the questionnaire Patient-Rated Wrist Evaluation (PRWE) [8], information about the study, additional questions regarding dexterity, problems with pronation/supination and previous injuries, and a consent form to fill out and return. After acceptance of participation, referrals for radiological follow-up were sent to the radiology department closest to the patient’s home address if the patient agreed to have new radiographs taken.

### Surgical and non-surgical treatment

Treatment was chosen by the attending orthopaedic surgeon. All patients were examined by plain radiographs with an anteroposterior and a lateral projection. Computed tomography was sometimes added. Patients who were treated non-surgically were immobilised in a below-elbow dorsal cast for approximately four weeks. Non-surgical treatment of the distal ulna fracture was also chosen in cases where the distal radius fracture was internally fixed, and in the remainder both the distal ulna and the distal radius were internally fixed. Fixation of the ulna fractures was accomplished through K-wires, screws or plates. All patients who received a surgical treatment were also postoperatively immobilised in a dorsal below-elbow cast for approximately 2 weeks.

### Classification

**Fig. 1:**
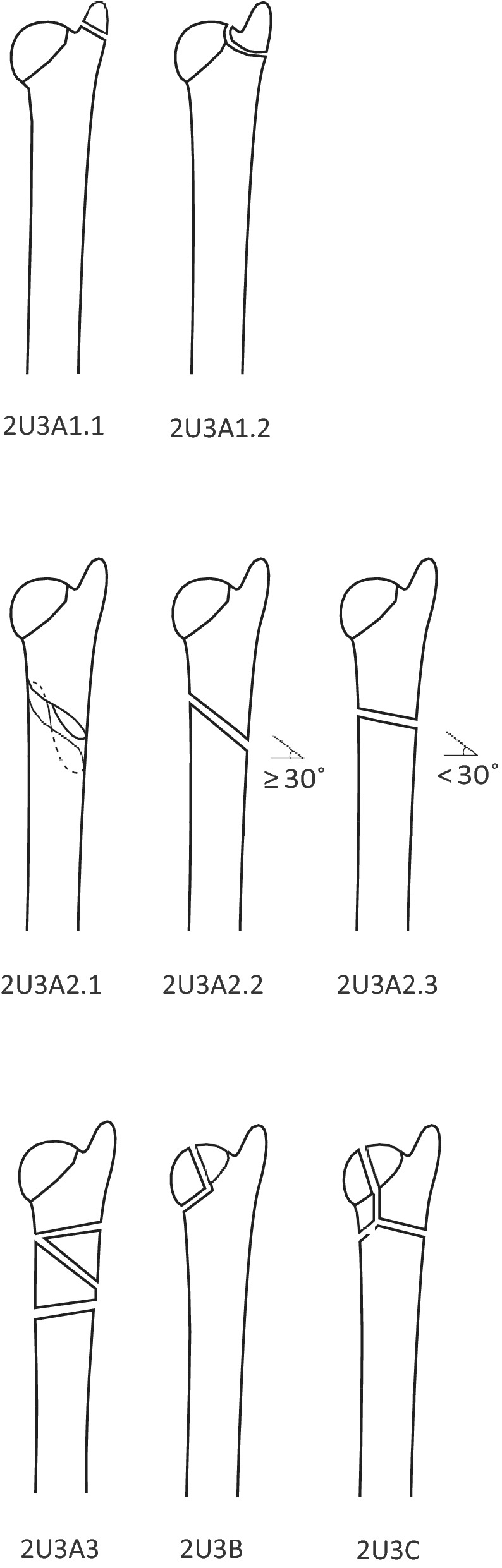
2018 AO/OTA fracture classification

The initial radiographs of the included fractures were passed on to two specialists in radiology who classified the fractures according to the 2018 AO/OTA classification [10]. In this classification, radius and ulna are coded as individual bones. The distal segment of the ulna is classified as 2U3 followed by: A for extra articular, B for partial articular, and C for complete articular fractures. 2U3A is further subdivided into 1 for styloid process [subdivided into (1) Tip and (2) Base], 2 for simple [subdivided into (1) Spiral, (2) Oblique, and (3) Transverse], and 3 for multifragmentary. This means that there are eight possible classes of distal ulna fractures (see Fig. 1). There are also universal modifiers that can be added to the end of the fracture code, for example impaction or displacement.

All fractures were classified using the initial radiographs, consisting of an anteroposterior and a lateral projection. The two radiologists classified the fractures independently, and in fractures where different classes had been selected the final decision regarding fracture class was made through consensus discussion. The initial radiographs were also reviewed for fracture displacement, which was defined as more than one cortical width, more than 1 mm step or more than 10° malangulation. The two radiologists had been specialists in radiology for 36 and 25 years, respectively.

#### Outcome measures

As primary outcome measure Patient-Rated Wrist Evaluation (PRWE) was used. PRWE is a self-administered questionnaire consisting of 15 questions divided into two subscales that assess both pain and function. The total score ranges from 0 (perfect wrist) to 100 (completely disabled and painful wrist). The Swedish version has been validated by two different research groups [11, 17]. In the case of incomplete or incorrect answers of the PRWE the interpretation was done according to the user manual for PRWE [7].

New radiographs were taken in all patients who agreed to this, with two projections, anteroposterior and lateral, of both the injured wrist and the contralateral wrist for comparison. All images were examined to determine; if the fracture had healed, if there was a remaining malunion of the ulna, if there were signs of osteoarthritis in DRUJ and/or in the radiocarpal and/or intercarpal and/or carpometacarpal joints, and also if there was a resulting difference of ulnar variance. All radiographs, both initial and at follow-up, were examined in 2020.

#### Statistics

The score of PRWE is presented as median (interquartile range, IQR). To compare median values the non-parametric SPSS Median test was used and to compare other variables, the chi-squared test was used. Significance level was set at 0.05.

## Results

### Patients

190 patients with 191 distal ulna fractures injured 2010–2014 were found eligible for follow-up and contacted. Ninety-six patients with 97 fractures agreed to participate in the study and filled out the PRWE, and 65 patients (66 fractures) also agreed to have their wrists radiographically examined. Commonly, the reasons for not wanting to go for new radiographs were old age and health issues in combination with the discomfort of an extra visit to the hospital.

Mean age at the time of injury was 63 years (SD 14.5). Seventeen were men and 79 were women. In 46% the injury affected the dominant wrist. Twenty-two patients had an isolated ulna fracture.

### Classification

Both radiologists found the AO/OTA 2018 classification difficult to use, in the way that a lot of fractures in our material did not fit easily into either of the eight classes. Sometimes a fracture was not considered to fit well into either and sometimes partially into several different classes. In 31% of the fractures a consensus discussion was needed for agreeing on the AO classification after different classes was initially suggested.

**Fig. 2:**
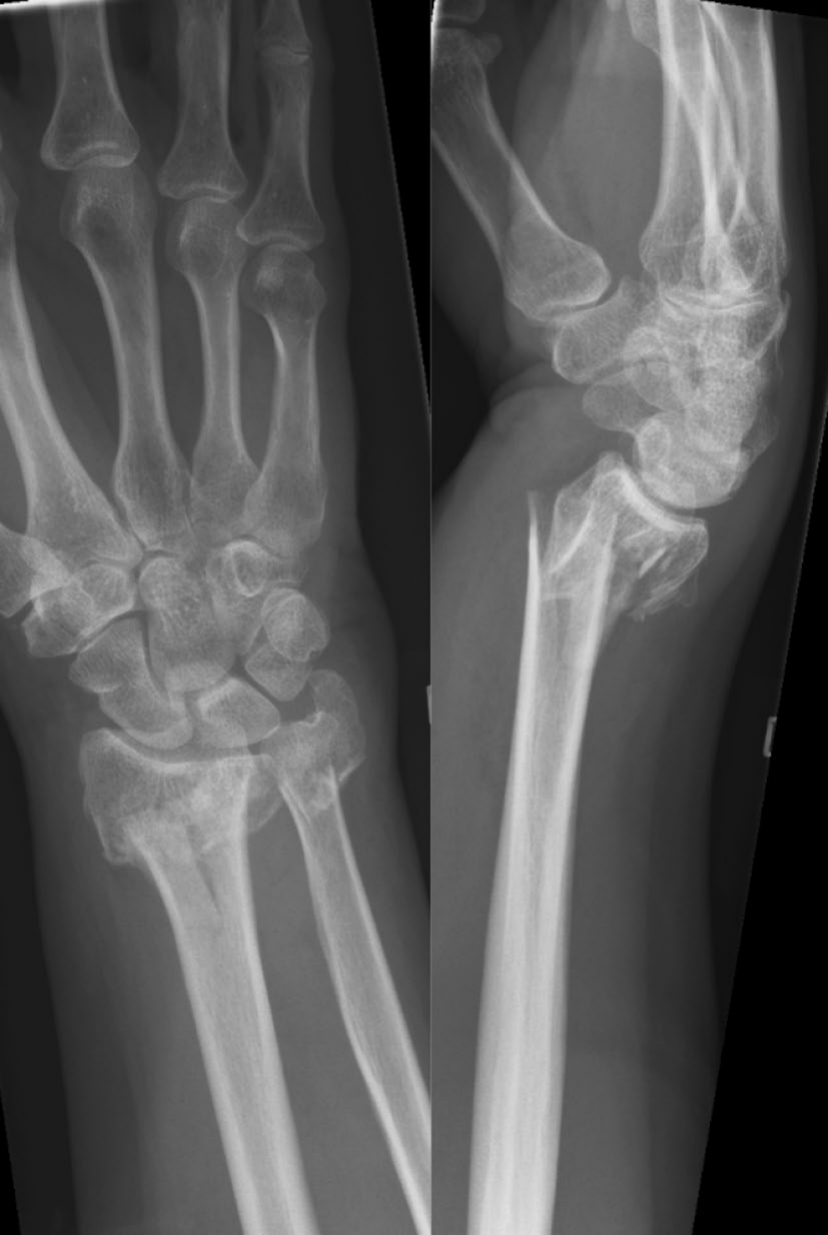
A typical fracture of AO class 2U3A2.3

In this series there was only one fracture each of class 2U3A1.2 (fracture of the styloid base) and of 2U3A2.1 (extra-articular spiral fractures) and none classified as 2U3B. Most common were extra-articular transverse fractures, 2U3A2.3, see Fig. 2, Table 1.

**Table 1:** Demographics and results divided by fracture class. PRWE (Patient-Rated Wrist Evaluation) scores presented as median (IQR)

	2U3A2.2 Extra-articular oblique (≥ 30°)	2U3A2.3 Extra-articular transverse (< 30°)	2U3A3 Extra-articular multifragmentary	2U3C Complete articular
Amount (*n*)	24	41	13	17
Age (mean, years)	62	64	65	63
Isolated (*n*)	7 (29%) (3 operated)	8 (20%) (2 operated)	6 (46%) (2 operated)	0
Ulna operated, (*n*)	11 (46%)	16 (39%)	7 (54%)	5 (29%)
Only radius operated, (*n*)	9 (38%)	13 (32%)	0	7 (41%)
Not operated at all, (*n*)	4 (17%)	12 (29%)	6 (46%)	5 (29%)
Follow-up radiographs (*n*)	16	30	7	11
Healed (*n*)	15 (94%)	29 (97%)	7 (100%)	11 (100%)
Remaining displacement (*n*)	2 (13%)	8 (27%)	1 (14%)	2 (18%)
Osteoarthritis DRUJ (*n*)	5 (31%)	15 (50%)	0	2 (18%)
Any carpal or DRUJ osteoarthritis (*n*)	9 (56%)	21 (70%)	1 (14%)	5 (45%)
Ulnaminus (*n*)	4 (25%)	4 (13%)	2 (29%)	3 (27%)
PRWE total	9.3 (30.3)	19 (36.8)	6 (34.3)	17.5 (33)
PRWE pain	5 (16)	10 (22)	4 (20)	10 (22)
PRWE function	5.8 (17.5)	7.5 (20.5)	2 (16.8)	6.5 (12.5)
PRWE Ulna operated	21.5 (60) (*n* = 11)	33 (27.3) (*n* = 16)*	7 (54) (*n* = 7)	17.5 (35.5) (*n* = 5)
PRWE Ulna not operated	7.5 (18.5) (*n* = 13)	10.5 (19.5) (*n* = 25)	1.5 (28.1) (*n* = 6)	13.5 (31.6) (*n* = 12)

*Indicates a significant difference with *p* < 0.05

### Treatment

Thirty-nine (40%) of the distal ulna fractures were treated with internal fixation, using K-wires, screws or plates. Out of the remaining 58 ulna fractures that were not surgically treated, 28 (29%) were not operated at all and instead treated with immobilisation in a below-elbow cast for approximately 4 weeks. In 30 cases (31%) only a concomitant fracture of the radius had undergone internal fixation. All operated isolated ulna fractures were fixed with plates and screws. All patients that received a surgical treatment were also immobilised in a below-elbow cast for approximately 2 weeks.

Details of the operative treatment are shown in Table 2.

**Table 2:** Operative treatment

	Amount, *n* (%)
K-wires	8 (20.5)
Screws	1 (2.5)
Plate and screws	30 (77)

### Outcome measures

Sixty-five patients (66 fractures) had radiographs taken at follow-up 5–7 years after the injury revealing that two fractures (3%) were not healed. One was never surgically treated while the other initially was fixed with a plate, but after multiple re-operations received a Sauvé-Kapandji procedure. Thirteen (20%) had a residual displacement defined as at least one cortical width, more than 1 mm intra articular step or 10° of malangulation. Out of these 77% had been treated non-operatively. Radiological signs of osteoarthritis in the DRUJ were found in 22 wrists (33%). In 36 (55%), osteoarthritis was seen somewhere in the radiocarpal, intercarpal or carpometacarpal joints.

All 96 included patients answered the PRWE. The median score of PRWE was 15 (IQR 33.5) and the maximum score for a single patient was 86. Extra-articular multifragmentary fractures had a PRWE score of 6 (IQR 34.3), extra-articular transverse fractures a score of 19 (IQR 36.8), extra-articular oblique fractures a score of 9.25 (IQR 30.3) and complete articular a score of 17.5 (IQR 33), respectively. There was no statistically significant difference in PRWE score between fracture classes.

The PRWE score for operated ulna fractures was 27.5 (IQR 36) as compared to the not operated with a PRWE of 7.75 (IQR 22), *p* = 0.01. See Table 3. When relating the operated and non-operated fractures to the AO classification, a significant difference in the PRWE score in favour of the non-operated was seen in class 2U3A2.3 (*p* = 0.001). No significant differences were seen in the other fracture classes, see Table 1*.* The fracture type most often (54%) treated by internal fixation was the extra-articular multifragmentary. Internal fixation was most infrequent for complete articular fractures, 29%.

**Table 3:** PRWE results, divided by treatment and by signs of osteoarthritis

	Operated ulna	Not operated ulna	*p*	Osteoarthritis	No osteoarthritis	*p*
PRWE total	27.5 (36)	7.75 (22)	*p* = 0.01*	22.75 (40.3)	7 (20.8)	*p* = 0.017*
PRWE pain	15 (20)	3.5 (13)	*p* = 0.025*	12 (23)	4 (15)	*p* = 0.059
PRWE function	11 (22.5)	2.75 (9.1)	*p* = 0.031*	9.25 (21.3)	2.5 (6.8)	*p* = 0.001*

*Indicates a significant difference with *p* < 0.05

The 41 transverse extra-articular fractures 2U3A2.3 were further examined based on initial displacement. Displaced fractures were significantly more often treated by internal fixation (*p* = 0.012) but there was no significant difference in the PRWE score between the displaced and the non-displaced fractures (*p* = 0.073) nor depending on a displaced fracture being operated or not (*p* = 0.21).

**Fig. 3:**
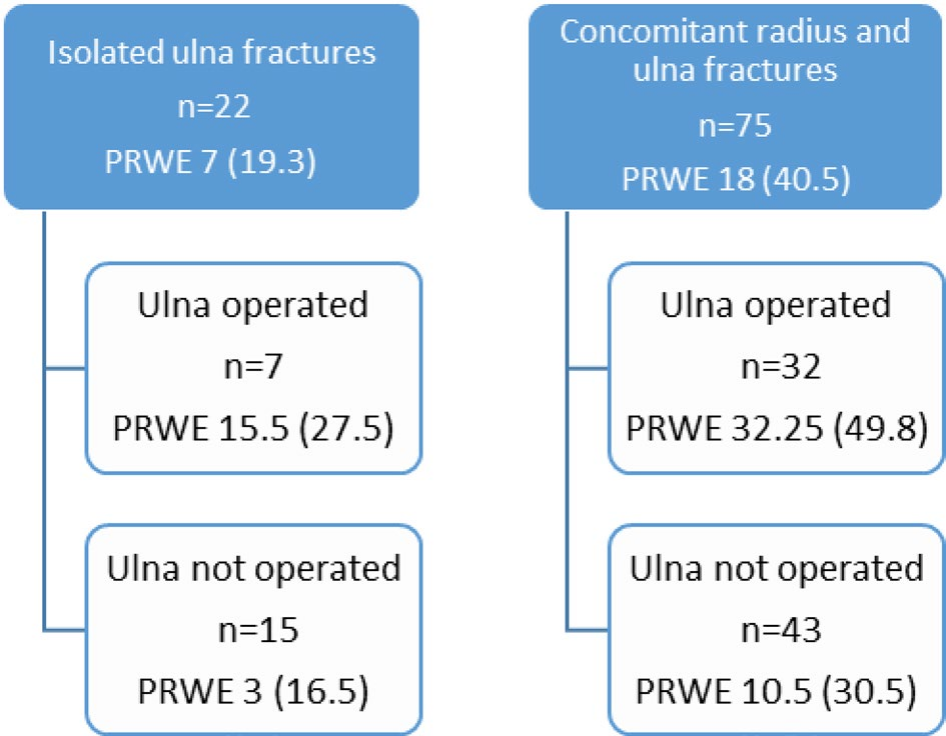
PRWE results comparing isolated ulna fractures with concomitant radius and ulna fractures

Patients with radiographic signs of osteoarthritis had a significantly worse PRWE score of 22.8 (IQR 40.3), compared to 7 (IQR 20.8), for patients without osteoarthritis, *p* = 0.017, see Table 4. The operated group had significantly more often osteoarthritis somewhere in the wrist (70%) compared to the non-operated group (45%), *p* = 0.047. There was no statistical difference regarding the presence of DRUJ osteoarthritis between the operated (39%) and the non-operated group (32%). The PRWE score was not related to initial displacement of the ulna fracture, a concomitant radius fracture, malunion, ulna variance or DRUJ osteoarthritis. Patients with isolated ulna fractures and patients with concomitant radius and ulna fractures are summarized in Fig. 3. The PRWE scores did not differ significantly between patients with isolated ulna fractures and those with a concomitant radius fractures and not between non-operated and operated isolated fractures.

**Table 4:** PRWE results divided by osteoarthritis or not

	Osteoarthritis	No osteoarthritis	*p*
PRWE total	22.75 (40.3)	7 (20.8)	*p* = 0.017*
PRWE pain	12 (23)	4 (15)	*p* = 0.059
PRWE function	9.25 (21.3)	2.5 (6.8)	*p* = 0.001*

*Indicates a significant difference with *p* < 0.05

## Discussion

The present study shows that distal ulna fractures almost always unite. As long as the radius is stable, ulna seems to heal regardless of the different treatment alternatives. The results, as assessed by the median PRWE, at follow-up 5–7 years after the injury was 15. This is comparable to the result after isolated distal radius fractures where it has been reported to be between 3 and 19 points [1, 3, 9, 11]. According to the median PRWE score for the patients in the present study fractures treated by internal fixation had a significantly worse result. When relating the PRWE results to the fracture types described by the recent AO classification this result was statistically significant for the extra-articular transverse fractures. These fractures, when having been operated, were associated with a higher PRWE score, both of function and pain. We do not know the selection process for the different treatments but the surgically treated fractures more often had an initial displacement and it may be that patients that had been subjected to a more severe injury, possibly with associated cartilage and soft tissue lesions, more often presented with displaced fractures. Such injuries are also likely more prone to develop secondary post traumatic arthritis, which in turn cause larger disability, demonstrated here by higher PRWE scores. Although the initial displacement apparently affected the choice of treatment, we could not prove that the displacement itself had led to a significantly worse result as measured by the PRWE and it cannot be ruled out that the addition of a surgical trauma has had a negative effect. Based on these findings it appears as if transverse extra-articular fractures do not benefit from surgical treatment.

Studies of distal ulna fractures are few. Sato et al. retrospectively reviewed 18 patients with combined distal radius and ulna fractures. In their series all metaphyseal ulna fractures had healed, nine had an excellent and one a good score according to the Gartland and Werley system and the mean DASH score was 4.1. They concluded that conservative treatment is acceptable for distal ulna fractures if the distal radius fracture is fixed with a volar locking plate [16]. In a prospective study of 24 patients with a distal ulna fracture and a concomitant radius fracture, Liang et al. [5] found one case of non-union, and one case of delayed union. They reported better radiographic appearance after surgical treatment and that most patients achieved a good or excellent score based on the Gartland–Werley demerit system. The limited number of patients and different group sizes did, however, not allow statistical analyses and guidelines for treatment [5]. A recent study used unplanned surgery after initial treatment as a measure of successful treatment. Out of 277 ulnar neck fractures with concomitant radius fractures 2.5% had an unplanned surgery. The indications were most often symptomatic implants and loss of fixation within 1 month. Factors associated with unplanned surgery were younger age, open fracture, multifragmented fracture and initial operative treatment. This study, however, did not investigate the final result or the functional outcome for the patients [14]. A retrospective study on 48 isolated distal ulna fractures showed similar results to ours, with no difference in healing time regardless of whether the fracture was surgically treated or not, but more associated injuries and more complications in the operated group had [18].

It has been previously shown that osteoarthritis can have an impact on the PRWE score. In a long-term follow-up of operated TFCC-injuries, patients who developed signs of osteoarthritis had a significantly worse score in the pain component of PRWE [12]. In the present study, a majority of the operated patients had signs of osteoarthritis in the DRUJ, radiocarpal, intercarpal or carpometacarpal joints. This could indicate more complex injuries in the operated patients, rather than the operation itself yielding an inferior result. When related to the AO classes, no significant differences were seen in the PRWE scores depending on the presence of osteoarthritis or not. This effect could, however, be due to the limited group sizes.

We found classification of distal ulna fractures with the 2018 AO/OTA system difficult to perform. Most often wrist radiographs are focused on the distal radius with only two views available, one anteroposterior and one lateral. From these images, the radiologists sometimes found it difficult to exactly determine the extent of the fracture system. Often the fractures in our material did not fit perfectly into either class or fit partially into several. This is highlighted by the large number of fractures needing a consensus discussion for classification. For a more accurate classification, a CT scan, potentially a cone beam CT (CBCT), could have been helpful. Another potential weakness of the AO classification is that the degree of displacement is not considered in the system.

A limitation of our study is that we have no information about the process of decision-making, the level of experience or number of orthopaedic surgeons who treated the patients, selected the type of treatment and performed the surgery. Another limitation is that description of the fractures according to the new AO classification resulted in very small numbers in some classes which made it impossible to determine if some fracture types had a worse prognosis.

The strengths of the study are that it represents one of the largest investigations of distal ulna fractures. The three hospitals in the county have agreed on a common treatment regime for wrist fractures and we, therefore, believe that all included patients were treated in a similar fashion. Our radiographic examinations and classifications were independently performed by two experienced specialists in radiology. In conclusion, we found the updated AO classification system difficult to use, especially if dependent only on standard radiographic views. The transverse extra-articular ulna fractures did not seem to benefit from internal fixation regardless if associated with a distal radius fracture or isolated.

## References

[1] Arora R, Gabl M, Gschwentner M, Deml C, Krappinger D, Lutz M (2009). A comparative study of clinical and radiologic outcomes of unstable colles type distal radius fractures in patients older than 70 years: nonoperative treatment versus volar locking plating. J Orthop Trauma.

[2] Biyani A, Simison AJ, Klenerman L (1995). Fractures of the distal radius and ulna. J Hand Surg.

[3] Grewal R, MacDermid JC, Pope J, Chesworth BM (2007). Baseline predictors of pain and disability one year following extra-articular distal radius fractures. Hand.

[4] Huang JI, Hanel DP (2012). Anatomy and biomechanics of the distal radioulnar joint. Hand Clin.

[5] Liang B, Lai JM, Murugan A, Chee KG, Sechachalam S, Foo TL (2015). Proposed guidelines for treatment of concomitant distal radius and distal ulna fractures. Hand Surg.

[6] Logan AJ, Lindau TR (2008). The management of distal ulnar fractures in adults: a review of the literature and recommendations for treatment. Strategies Trauma Limb Reconstr.

[7] MacDermid JC (2011) The patient-rated wrist evaluation (PRWE) user manual. McMaster University, Hamilton, Ontario, Canada

[8] MacDermid JC, Turgeon T, Richards RS, Beadle M, Roth JH (1998). Patient rating of wrist pain and disability: a reliable and valid measurement tool. J Orthop Trauma.

[9] MacFarlane RJ, Miller D, Wilson L (2015). Functional outcome and complications at 2.5 years following volar locking plate fixation of distal radius fractures. J Hand Microsurg.

[10] Meinberg EG, Agel J, Roberts CS, Karam MD, Kellam JF (2018). Fracture and dislocation classification compendium-2018. J Orthop Trauma.

[11] Mellstrand Navarro C, Ponzer S, Tornkvist H, Ahrengart L, Bergstrom G (2011). Measuring outcome after wrist injury: translation and validation of the Swedish version of the patient-rated wrist evaluation (PRWE-Swe). BMC Musculoskelet Disord.

[12] Moloney M, Farnebo S, Adolfsson L (2018). 20-Year outcome of TFCC repairs. J Plast Surg Hand Surg.

[13] Moloney M, Farnebo S, Adolfsson L (2020). Incidence of distal ulna fractures in a Swedish county: 74/100,000 person-years, most of them treated non-operatively. Acta Orthop.

[14] Ozkan S, Fischerauer SF, Kootstra TJM, Claessen F, Ring D (2018). Ulnar neck fractures associated with distal radius fractures. J Wrist Surg.

[15] Ring D, McCarty LP, Campbell D, Jupiter JB (2004). Condylar blade plate fixation of unstable fractures of the distal ulna associated with fracture of the distal radius. J Hand Surg.

[16] Sato K, Murakami K, Mimata Y, Numata N, Shiraishi H, Doita M (2018). Conservative treatment of distal ulna metaphyseal fractures associated with distal radius fractures in elderly people. Orthop Traumatol Surg Res.

[17] Wilcke MT, Abbaszadegan H, Adolphson PY (2009). Evaluation of a Swedish version of the patient-rated wrist evaluation outcome questionnaire: good responsiveness, validity, and reliability, in 99 patients recovering from a fracture of the distal radius. Scand J Plast Reconstr Surg Hand Surg.

[18] Williams EA, Friedrich JB (2011). Retrospective analysis demonstrates no advantage to operative management of distal ulna fractures. Hand.

